# Supplementary figures and images for: Phylogenomics indicates the “living fossil” Isoetes diversified in the Cenozoic
Source: PLoS One. 2020 Jun 18;15(6):e0227525. doi: 10.1371/journal.pone.0227525 (PMC7302493; doi:10.1371/journal.pone.0227525)

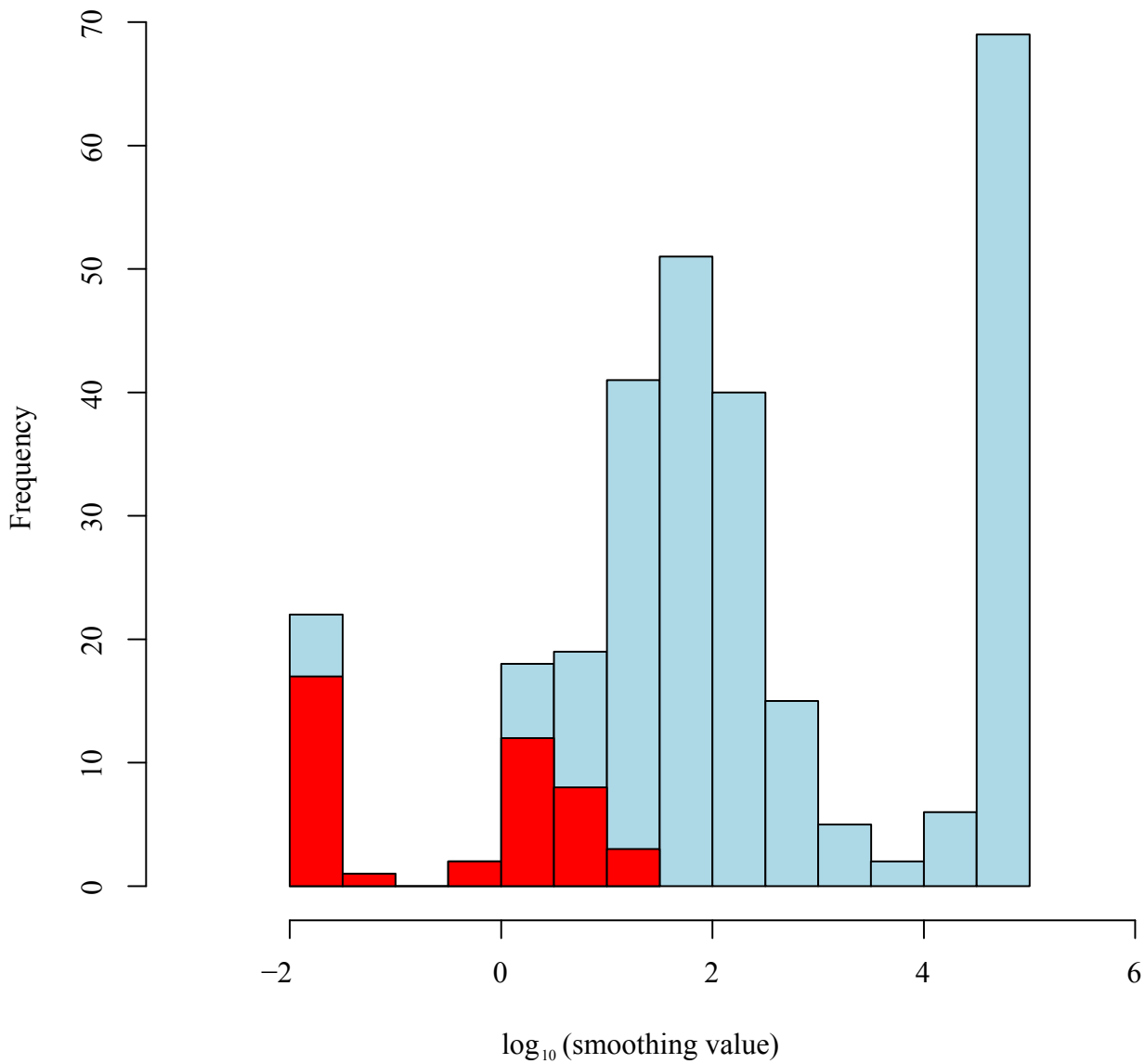

Supplement: S1 Fig — Histogram of optimum smoothing values in r8s identified by cross validation for individual nuclear genes. Proportion of genes for each smoothing value that fail gradient checks are highlighted in red. (PDF) [file pone.0227525.s001.pdf]

**0.01**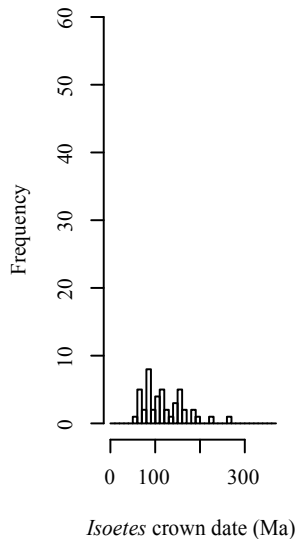**0.1**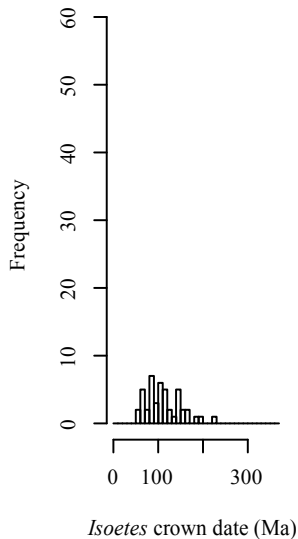**1**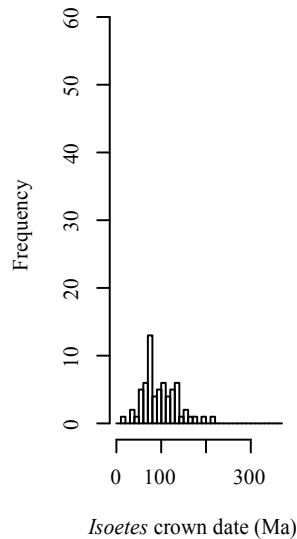**10**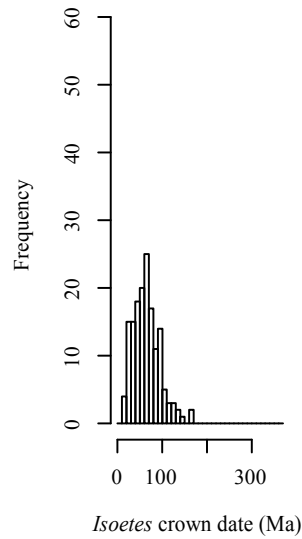**100**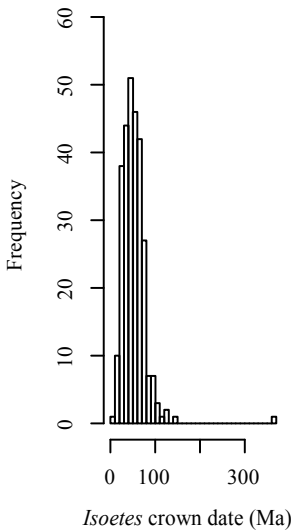**1000**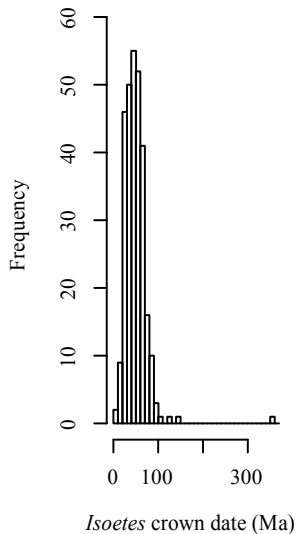**10000**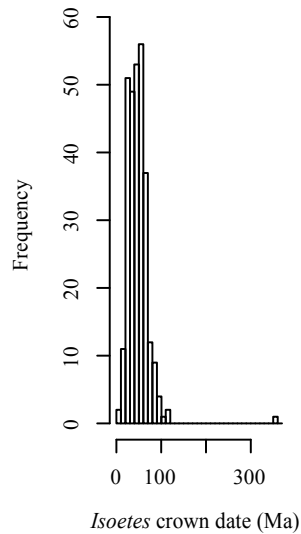**Optimum**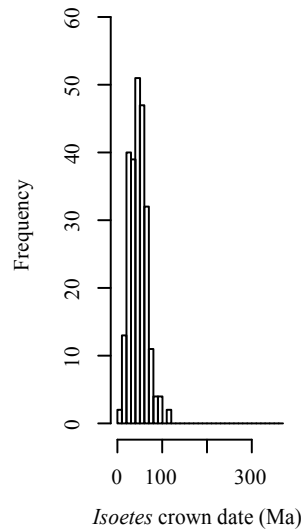

Supplement: S2 Fig — Histograms showing estimated Isoetes crown group dates for individual nuclear genes in r8s that pass gradient checks for a range of assigned smoothing values, and the histogram of estimates where each gene is assigned its optimum smoothing value based on cross validation (final panel). (PDF) [file pone.0227525.s002.pdf]

**0.01**

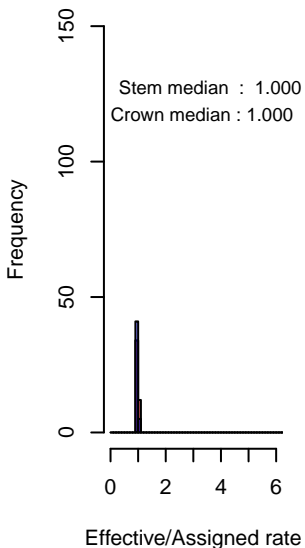

**0.1**

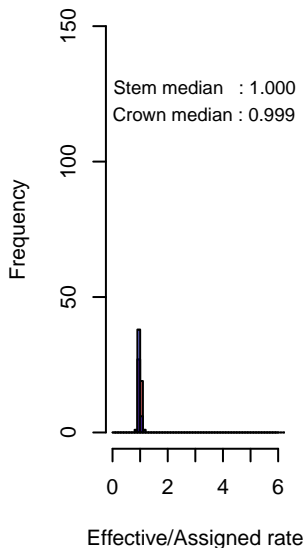

**1**

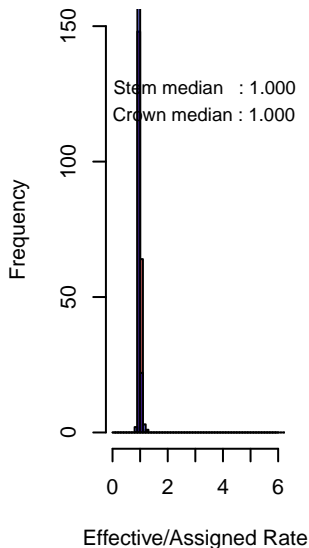

**10**

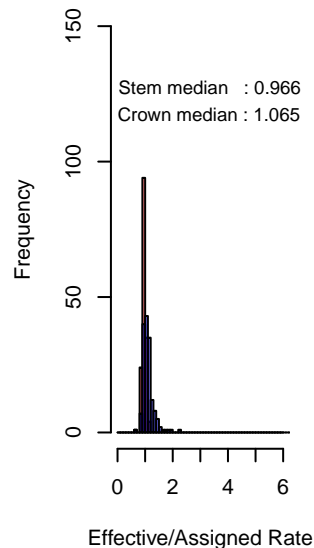

**100**

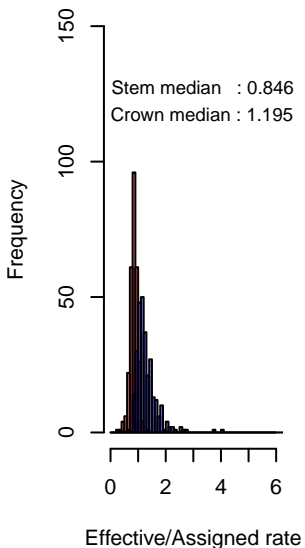

**1000**

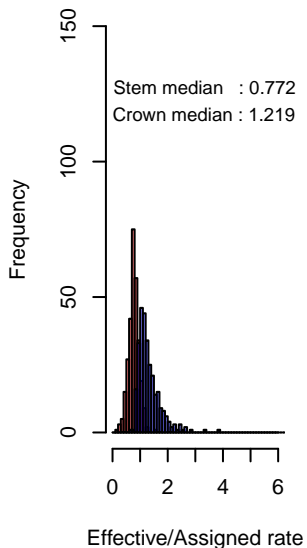

**10000**

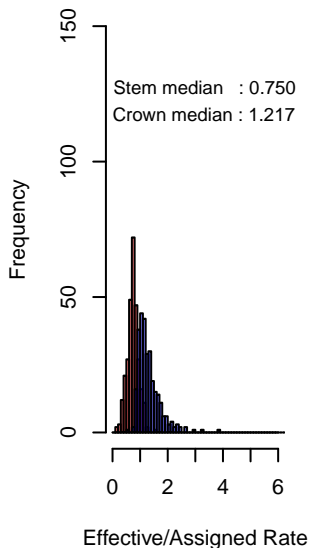

**Optimum**

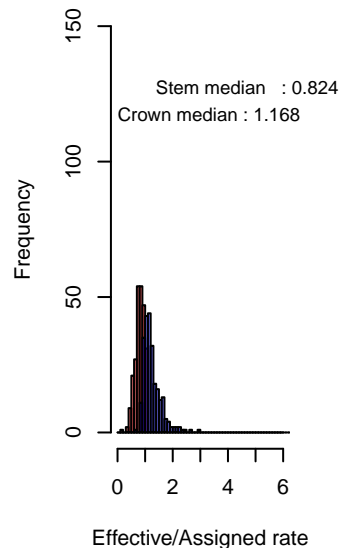

Supplement: S3 Fig — Histograms of the ratio of effective vs. assigned branch rates for the stem (red) and average value for crown (blue) branches of Isoetes for individual nuclear genes in r8s that passed gradient checks for a range of assigned smoothing values, and the histogram of estimates where each gene is assigned its optimum smoothing value based on cross validation (final panel). Median values are displayed in the top righthand corner of each panel. (PDF) [file pone.0227525.s003.pdf]
