# Supplementary material for: Phylogenomics indicates the “living fossil” Isoetes diversified in the Cenozoic
Source: PLoS One. 2020 Jun 18;15(6):e0227525. doi: 10.1371/journal.pone.0227525 (PMC7302493; doi:10.1371/journal.pone.0227525)
Supplement: S1 Table — Published with the permission of the Board of Trustees of the Royal Botanic Gardens, Kew. (DOCX) [file pone.0227525.s004.docx]

| **Recorded species name** | **Collection location** | **Kew DNA Bank ID** | **Specimen ID** |
| --- | --- | --- | --- |
| *Isoetes nuttallii* | Alaska, USA | 46188 | Eyerdam 3373 |
| *Isoetes andicola* | Lima, Peru | 46189 | K000374086/7 |
| *Isoetes humilior* | Australian Capital Territory, Australia | 46214 | H2016/0029759 |
| *Isoetes elatior* | Tasmania, Australia | 46215 | K34597.000 |
| *Isoetes coromandelina* | Rajasthan, India | 46160 | H2016/0029745 |
