# Supplementary material for: Phylogenomics indicates the “living fossil” Isoetes diversified in the Cenozoic
Source: PLoS One. 2020 Jun 18;15(6):e0227525. doi: 10.1371/journal.pone.0227525 (PMC7302493; doi:10.1371/journal.pone.0227525)
Supplement: S2 Table — (DOCX) [file pone.0227525.s005.docx]

| **Species** | **Source** | **Reference** |
| --- | --- | --- |
| *Amborella trichopoda* | NCBI: NC_005086 | 1 |
| *Ananas comosus* | NCBI: NC_026220 | 2 |
| *Aneura mirabilis* | NCBI: NC_010359 | 3 |
| *Ceratophyllum demersum* | NCBI: EF614270 | 4 |
| *Diploterygium glaucum* | NCBI: NC_024158 | 5 |
| *Ginkgo biloba* | NCBI: NC_016986 | Unpublished |
| *Huperzia lucidula* | NCBI: NC_006861 | 6 |
| *Isoetes flaccida* | NCBI: NC_014675 | 7 |
| *Lygodium japonicum* | NCBI: KF225593 | 5 |
| *Marchantia polymorpha* | NCBI: NC001319 | 8 |
| *Nuphar advena* | NCBI: DQ354691 | 9 |
| *Nymphaea alba* | NCBI: AJ627251 | 10 |
| *Osmundastrum cinnamomeum* | NCBI: NC_024157 | 5 |
| *Physcomitrella patens* | NCBI: AP005672 | 11 |
| *Pinus thunbergii* | NCBI: D17510 | 12 |
| *Plantago maritima* | NCBI: NC_028519 | 13 |
| *Plantago media* | NCBI: NC_028520 | 13 |
| *Pteridium aquilinum* | NCBI: NC_014348 | 14 |
| *Ranunculus macranthus* | NCBI: NC_008796 | 9 |
| *Selaginella mollendorffii* | NCBI: HM173080 | 15 |
| *Selaginella uncinata* | NCBI: AB197035 | 16 |
| *Sesamum indicum* | NCBI: JN637766 | 17 |
| *Syntrichia ruralis* | NCBI: FJ546412 | 18 |
| *Veronica nakaiana* | NCBI: NC_031153 | 19 |

**References:**

1. Goremykin,V.V., Hirsch-Ernst,K.I., Wolfl,S. and Hellwig,F.H. Analysis of the *Amborella trichopoda* Chloroplast Genome Sequence Suggests That Amborella Is Not a Basal Angiosperm. Mol. Biol. Evol. 2003;20(9): 1499-1505.

2. Nashima K, Terakami S, Nishitani C, Kunihisa M, Shoda M, Takeuchi M, Urasaki N, Tarora K, Yamamoto T, Katayama H. Complete chloroplast genome sequence of pineapple (*Ananas comosus*). Tree Genet. Genomes. 2015;11(3): 60.

3. Wickett,N.J., Zhang,Y., Hansen,S.K., Roper,J.M., Kuehl,J.V., Plock,S.A., Wolf,P.G., DePamphilis,C.W., Boore,J.L. and Goffinet,B. Functional gene losses occur with minimal size reduction in the plastid genome of the parasitic liverwort *Aneura mirabilis.* Mol. Biol. Evol. 2008;25(2): 393-401.

4. Moore,M.J., Bell,C.D., Soltis,P.S. and Soltis,D.E. Using plastid genome-scale data to resolve enigmatic relationships among basal angiosperms. Proc. Natl. Acad. Sci. U.S.A. 2007;104(49): 19363-8.

5. Kim,H.T., Chung,M.G. and Kim,K.J. Chloroplast Genome Evolution in Early Diverged Leptosporangiate Ferns. Mol. Cells. 2014;37(5): 372.

6. Li,X., Li,Q., Lin,X., Hu,Z. and Chen,S. High-throughput multiplex sequencing reveals the prospect of chloroplast genomes as a plant super-barcode.

7. Wolf,P.G., Karol,K.G., Mandoli,D.F., Kuehl,J., Arumuganathan,K.,Ellis,M.W., Mishler,B.D., Kelch,D.G., Olmstead,R.G. and Boore,J.L. The first complete chloroplast genome sequence of a lycophyte, *Huperzia lucidula* (Lycopodiaceae). Gene. 2005;350(2): 117-28.

8. Shimada,H. and Sugiura,M. Fine structural features of the chloroplast genome: comparison of the sequenced chloroplast genomes. Nucleic Acids Res. 1991;19 (5): 983-95.

9. Raubeson LA, Peery R, Chumley TW, Dziubek C, Fourcade HM, Boore JL, Jansen RK. Comparative chloroplast genomics: analyses including new sequences from the angiosperms Nuphar advena and Ranunculus macranthus. BMC genomics. 2007;8(1): 174.

10. Goremykin VV, Hirsch-Ernst KI, Wölfl S, Hellwig FH. The chloroplast genome of Nymphaea alba: whole-genome analyses and the problem of identifying the most basal angiosperm. Mol. Biol. Evol. 2004;21(7): 1445-54.

11. Sugiura C, Kobayashi Y, Aoki S, Sugita C, Sugita M. Complete chloroplast DNA sequence of the moss Physcomitrella patens: evidence for the loss and relocation of rpoA from the chloroplast to the nucleus. Nucleic Acids Research. 2003;31(18): 5324-31.

12. Tsudzuki J, Ito S, Tsudzuki T, Wakasugi T, Sugiura M. A new gene encoding tRNA Pro (GGG) is present in the chloroplast genome of black pine: a compilation of 32 tRNA genes from black pine chloroplasts. Curr. Genet. 1994;26(2): 153-8.

13. Zhu A, Guo W, Gupta S, Fan W, Mower JP. Evolutionary dynamics of the plastid inverted repeat: the effects of expansion, contraction, and loss on substitution rates. New Phytol. 2016;209(4): 1747-56.

14. Der JP. Genomic Perspectives on Evolution in Bracken Fern. Dissertation. 2010. Utah State University, Departmnet of Biology. 2010.

15. Banks,J.A., Nishiyama,T., Hasebe,M., Bowman,J.L., Gribskov,M., de Pamphilis C., et al. The *Selaginella* genome identifies genetic changes associated with the evolution of vascular plants. Science. 201;332(6032): 960-3.

16. Tsuji S, Ueda K, Nishiyama T, Hasebe M, Yoshikawa S, Konagaya A, Nishiuchi T, Yamaguchi K. The chloroplast genome from a lycophyte (microphyllophyte), *Selaginella uncinata*, has a unique inversion, transpositions and many gene losses. Journal of plant research. J. Plant Res. 2007;120(2): 281-90.

17. Yi DK, Kim KJ. Complete chloroplast genome sequences of important oilseed crop *Sesamum indicum* L. PloS one. 2012;7(5): e35872.

18. Oliver MJ, Murdock AG, Mishler BD, Kuehl JV, Boore JL, Mandoli DF, Everett KD, Wolf PG, Duffy AM, Karol KG. Chloroplast genome sequence of the moss *Tortula ruralis*: gene content, polymorphism, and structural arrangement relative to other green plant chloroplast genomes. BMC genomics. 2010;11(1): 143.

19. Choi KS, Chung MG, Park S. The complete chloroplast genome sequences of three Veroniceae species (Plantaginaceae): comparative analysis and highly divergent regions. Front. Plant Sci. 2016;7: 355.
