# Supplementary material for: Phylogenomics indicates the “living fossil” Isoetes diversified in the Cenozoic
Source: PLoS One. 2020 Jun 18;15(6):e0227525. doi: 10.1371/journal.pone.0227525 (PMC7302493; doi:10.1371/journal.pone.0227525)
Supplement: S3 Table — See main text for references. (DOCX) [file pone.0227525.s006.docx]

| **Species** | **Data type** | **Source** |
| --- | --- | --- |
| *Ananas comosus* | Assembly (v3.0) | Phytozome [84] |
| *Ceratodon purpurea* | Assembly | [83] |
| *Huperzia lucidula* | Reads | [86] |
| *Huperzia squarrosa* | Reads | [86] |
| *Isoetes sinensis* | Reads | SRA: SRR1648119 [85] |
| *Lygodium japonicum* | Assembly (v1.0) | <http://bioinf.mind.meiji.ac.jp/kanikusa> [82] |
| *Pinus pinaster* | Assembly (v3.0) | <http://www.scbi.uma.es/sustainpinedb/> [81] |
| *Pteridium aquilinum* | Assembly | [80] |
