# Supplementary material for: Phylogenomics indicates the “living fossil” Isoetes diversified in the Cenozoic
Source: PLoS One. 2020 Jun 18;15(6):e0227525. doi: 10.1371/journal.pone.0227525 (PMC7302493; doi:10.1371/journal.pone.0227525)
Supplement: S4 Table — (DOCX) [file pone.0227525.s007.docx]

| **Constraint** | **Taxon used** | **Taxon Age** | **Constraint value** | **Strata/Locality** | **Phylogenetic placement** | **Publication** |
| --- | --- | --- | --- | --- | --- | --- |
| *Crown Isoetopsida* (lower limit) | *Otzinachsonia beerboweri* | Famenian | 358 | Red Hill outcrop, Pennsylvania, USA | *Isoetales* | 94 |
| *Crown embryophyta* (lower limit) | *Baragwanthia longifolia* | Ludlow | 421 | Yea, Victoria, Australia | *Tracheophyta* | 101 |
| *Crown embryophyta* (upper limit) | Cryptospores | NA | 485 | NA | *Embryophyta* | 100 |
