# Supplementary material for: Phylogenomics indicates the “living fossil” Isoetes diversified in the Cenozoic
Source: PLoS One. 2020 Jun 18;15(6):e0227525. doi: 10.1371/journal.pone.0227525 (PMC7302493; doi:10.1371/journal.pone.0227525)
Supplement: S5 Table — Linear models in R (using the lm function) are used to identify the relationship between a number of alignment properties for individual nuclear genes and the resultant predicted dates in r8s and BEAST. Significant p-values (<0.05) are highlighted in bold. (DOCX) [file pone.0227525.s008.docx]

| **Relationship** | **BEAST gradient** | **BEAST p-value** | **BEAST R value** | **r8s gradient** | **r8s p-value** | **r8s R^2^ value** |
| --- | --- | --- | --- | --- | --- | --- |
| Average *Isoetes* alignment completeness | 0.291 | **0.0007** | 0.042 | 0.4 | **7.4E-05** | 0.059 |
| Average genome  skimming sample  completeness | 0.329 | **0.0018** | 0.036 | 0.35 | **0.0045** | 0.029 |
| *I. coromandelina*  alignment completeness | 0.355 | **0.00047** | 0.047 | 0.414 | **0.0005** | 0.049 |
| Average alignment  completeness | 0.16 | **0.038** | 0.014 | 0.25 | **0.0051** | 0.027 |
| Percentage of polymorphic sites | -0.087 | 0.22 | 0.0022 | -0.056 | 0.495 | -0.0022 |
| Percentage of phylogenetically informative sites | -0.045 | 0.616 | -0.0031 | 0.06 | 0.53 | -0.025 |
| Percentage completeness of *Selaginella* | 0.11 | **0.029** | 0.016 | 0.16 | **0.0071** | 0.0254 |
| Average completeness of  *I. sinensis* and *I. lacustris* | 0.09 | 0.092 | 0.0076 | 0.152 | **0.016** | 0.02 |
