# Supplementary material for: Phylogenomics indicates the “living fossil” Isoetes diversified in the Cenozoic
Source: PLoS One. 2020 Jun 18;15(6):e0227525. doi: 10.1371/journal.pone.0227525 (PMC7302493; doi:10.1371/journal.pone.0227525)
Supplement: S6 Table — Branch lengths from maximum likelihood phylogenies generated using RaxML (GTR+G+I model) for (i) all three markers used in the study, ii) the atpB-rbcL intergenic spacer removed, (iii) nrITS removed (iii) or iv) both the atpB-rbcL spacer and nrITS removed. (DOCX) [file pone.0227525.s009.docx]

| Analysis | Average tip to *Isoetes* crown branch length (substitutions per site) | Stem length (substitutions per site) |
| --- | --- | --- |
| All *rbcL* + *Isoetes* *nrITS* + Isoetes *atpB-rbcL* intergenic spacer | 0.042 | 0.053 |
| All *rbcL* + Isoetes *nrITS* | 0.054 | 0.067 |
| All *rbcL* + Isoetes *atpB-rbcL* intergenic spacer | 0.016 | 0.035 |
| All *rbcL* | 0.0084 | 0.033 |
